# A Retrospective Evaluation of the Safety of Combining Helium-Based Plasma Radiofrequency Technology With Ultrasound-Assisted Lipoabdominoplasty

**DOI:** 10.1093/asjof/ojae116

**Published:** 2025-03-04

**Authors:** Paul Vanek

## Abstract

**Background:**

Helium plasma radiofrequency (RF; Renuvion; Apyx Medical, Clearwater, FL) is cleared for use in the coagulation/contraction of subcutaneous soft tissue, in body contouring, and to address loose skin in the neck and submental region. The device instantly heats target tissue to >85 °C, causing rapid protein coagulation within 0.04 s, resulting in tissue contraction. The use of multiple energy-based devices in lipoabdominoplasty is controversial.

**Objectives:**

To assess the safety of helium plasma RF as an adjunct to lipoabdominoplasty utilizing ultrasound-assisted liposuction (UAL; VASER, Solta Medical, Bothell, WA).

**Methods:**

In this retrospective, single-center study, medical records for patients who underwent UAL and abdominoplasty with or without helium-based plasma RF as an adjunct for subdermal coagulation between October 2017 and March 2023 were reviewed. Primary outcomes included significant and nonsignificant adverse events (AEs). Univariate and multivariate analyses were used to identify any risk factors for AEs.

**Results:**

A total of 40 patients treated with lipoabdominoplasty and helium plasma RF and 37 patients treated with lipoabdominoplasty alone were included in the analysis. Overall, no significant difference between groups was detected for the occurrence of significant AEs (*P* = .628).

**Conclusions:**

In this study, it is indicated that the helium plasma RF device may be a safe adjunct for UAL lipoabdominoplasty. When used for subdermal coagulation in the abdominal area, the device does not appear to increase the incidence of serious AEs or introduce risk to the flap.

**Level of Evidence: 3 (Therapeutic):**

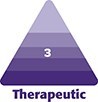

In 2022, liposuction was the most commonly performed surgical aesthetic procedure, with >400,000 individual procedures performed in the United States,^1^ representing >$1.1 billion in revenue. In the same year, abdominoplasty was the third most popular surgical procedure, with >207,000 procedures performed.^1^ When performed together as lipoabdominoplasty, patients can reap both aesthetic and functional benefits.^2^ Traditionally, liposuction performed in this setting is suction-assisted or power-assisted; however, multiple devices have been developed to facilitate fat removal, including laser-assisted lipolysis, radiofrequency (RF)-assisted lipolysis, and ultrasound-assisted liposuction (UAL). Although all of these devices have been reported to improve skin retraction and aesthetic outcomes for liposuction/lipoabdominoplasty,^3-5^ the use of thermal energy in this setting remains somewhat controversial, because the safety profile of helium plasma RF as an adjuvant in the abdominoplasty flap has not been characterized in the comparative literature.^6-8^

For most patients, the removal of skin and excess fat is insufficient to yield the desired result of a more sculpted appearance. Although surgical procedures tighten the skin through tissue removal, skin quality is not addressed. In clinical practice where UAL is used, significant tissue contraction and reduced blood loss can be achieved, compared with power-assisted liposuction;^5^ however, most patients desire more contraction than can be achieved with UAL alone. In order to fully address residual skin laxity, additional tissue coagulation and contraction beyond that which is achievable with these devices is often needed. Additional contraction is generally achieved through delivery of thermal energy to subcutaneous tissues by stand-alone heating devices. These devices may include technologies such as RF-based techniques or micro-focused ultrasound with visualization.^5^ However, these devices are somewhat limited because they rely on bulk heating of tissues, which requires that all tissues in the area be heated until the target tissue reaches the desired temperature of 65 °C.^9-16^ At 65 °C, maximal contraction occurs after 120 s,^17^ making treatment with these devices time-consuming, especially for a large area like the abdomen.

In the current study, the author assessed safety outcomes for patients treated with UAL liposuction (VASER, Solta Medical, Bothell, WA) in combination with helium-based plasma RF in the whole abdominoplasty flap (Renuvion; Apyx Medical, Clearwater, FL).^18,19^ Unlike bulk-heating methods, helium plasma RF is able to directly heat subcutaneous fascia and septal connective tissues as the device electrode is passed through the subcutaneous layer. The device's electrode is energized by RF, and when helium gas is passed over the electrode, it generates helium plasma and heat. In addition, a portion of the RF energy passes through the plasma beam to adjacent tissues, also generating heat. As the device is passed through the subcutaneous plane, the plasma beam “connects” to tissues that represent the path of least resistance and is constantly finding a new path, resulting in a quickly moving beam that treats tissues in multiple directions. Tissues surrounding the device electrode are instantly heated to temperatures >85 °C, which must be maintained for only 0.04 s to achieve complete protein coagulation.^17^ Importantly, the surrounding tissue cools quickly, and surface temperatures increase by 3.6 °C or less.^20,21^ The device is cleared by the United States FDA for the subcutaneous delivery of RF energy and/or helium plasma in which coagulation/contraction of soft tissue is needed, and more specifically for use in the neck and submental area to improve the appearance of lax skin and for use in the body for aesthetic body contouring.^18^ The limited use of UAL and abdominoplasty has been characterized in a small study in which it has been shown that there is no increased risk of adverse events (AEs) compared with the addition of limited-use power-assisted liposuction.^6^ The author has used helium plasma RF in the entire abdominoplasty flap for several years to improve outcomes in their lipoabdominoplasty patients and gain further improvement beyond that achieved with UAL, while also maintaining the excellent safety profile of UAL treatment alone.^5^ In order to understand whether the use of helium plasma RF in conjunction with UAL and abdominoplasty had an effect on safety outcomes, this real-word patient cohort was assessed as part of a retrospective review.

## METHODS

### Study Design

In this retrospective, single-center, single-surgeon study, medical records for patients who underwent UAL abdominoplasty with or without helium-based plasma RF as an adjunct for subdermal coagulation between October 2017 and March 2023 were reviewed. All patients who received lipoabdominoplasty at the site between October 2017 and March 2023 were included in the study. All patients were offered helium plasma RF, and those patients who elected to undergo the treatment along with their lipoabdominoplasty were included in the lipoabdominoplasty with helium plasma RF study group. Both groups were treated throughout the study period; groups were not treated as individual cohorts in sequence (ie, 1 group and then the other). All patients were 18 years of age or older and were treated in the abdominal area within the entire abdominoplasty flap, but they could also be treated in other areas of the body.

This study was approved by Sterling IRB, Atlanta, GA, and all procedures were conducted in accordance with the 1964 Declaration of Helsinki and its later amendments or comparable ethical standards.

### Primary Outcomes

The study's primary outcome measures included an analysis of the procedure and demographic information by group (UAL with helium plasma RF vs ultrasound-assisted lipoabdominoplasty alone) as well as an analysis of lipoabdominoplasty-related AEs. AEs related to concurrent procedures (eg, breast augmentation) were not included in the analysis. In addition, expected treatment effects (eg, edema and bruising) and systemic events (eg, anemia or obstipation) were not included. AEs requiring a subsequent in-office procedure, hospitalization, or a home health or emergency department (ED) visit were categorized as significant. All other AEs were categorized as nonsignificant.

### Statistical Analysis

*T* test or Fisher's exact test were used to assess differences between groups for continuous or categorical data, respectively. A univariate logistic regression model examined whether the amount of skin or the number of concomitant procedures impacted the probability of having a significant AE for a patient. Multivariate logistic regression models explored the predictors of a patient having a significant AE by impact of treatment group, age, BMI, smoking history, history of significant weight loss, amount of skin removed, and number of concomitant procedures.

### Treatment Methodology

**Figure 1.**
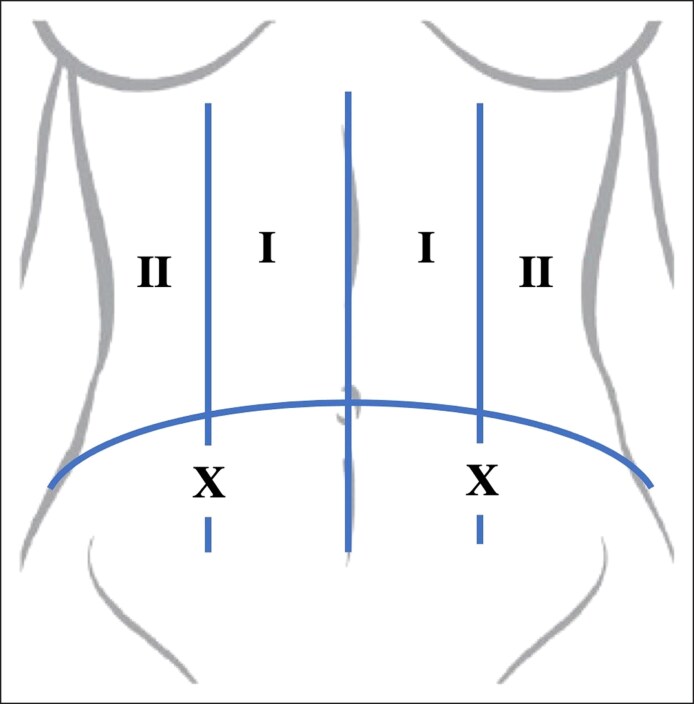
The author focuses on the abdomen in 2 zones: Zone I and Zone II. Zone numbers are indicated, and zones are demarcated with solid lines. The “X” on each side indicates the entry point for the ultrasound-assisted liposuction and helium plasma radiofrequency probe, which is later incorporated in the resection area for abdominoplasty.

Lipoabdominoplasty procedures were completed in a standardized manner. The patients were treated with the same case sequence in each arm: tumescent, UAL administrations, helium plasma RF, then surgical elevation of the skin flap. In some cases, UAL was also applied to other areas of the body such as the flanks, mons, back, axilla, and so on, on the basis of the patient treatment plan. Synchronous breast surgery was also performed in some patients, with the abdomen domain management performed last in case sequencing. The patient was marked in a standing position, with a single entry site on each side marked for probe access for tumescent; UAL administration; and helium plasma RF (Figure 1). The entry site was marked in the midclavicular line in the lateral border of the rectus, but within the domain of skin to be resected for the abdominoplasty. Treatment Zones I and II were also marked.

General endotracheal anesthesia was used for all patients. A super wet technique was employed for tumescent using a mixture 1 L lactated ringers solution, 10 cc of 0.5% bupivacaine and 1:1000 epinephrine, and 500 mg of tranexamic acid. The patients were tumesced with 420 to 500 mL total in Zone 1 for both areas and 430 to 565 mL per zone in Zone II areas. Ten minutes after the administration of tumescent, UAL and helium plasma RF were administered. UAL was applied first to Zone 2 (settings were 15-17 min, 70-80 V, 3 or 5 grooves, with between 440 and 620 cc of fat removed with a 4.6 vented cannula). For Zone I, settings were 9 to 10 min, 40 V, 2 grooves, with no fat removed from that area. In the helium plasma RF group, following UAL, helium plasma RF was evenly applied across the abdominoplasty flap in the subcutaneous and subdermal plane of the abdomen, utilizing the same incision points for 6 passes at each incision point in Zones I and II. There was no hollow cannula treatment in Zone I. An example of the treatment technique is provided in Video. Device settings were 80% power (range, 60%-100%), 2.5 L/min of helium flow, and 7 and 10 kJ of energy delivered in Zones 1 and 2, respectively. Following the application of helium plasma RF, residual helium gas and fluid were evacuated through aspiration and manual expression. The UAL/helium plasma RF treatment ports were incorporated into the resection area of lipoabdominoplasty.

Next, a standard abdominoplasty incision was performed with a 10-blade knife, carried down to the fascia. The abdominal flap was then elevated to the costal margin using low-current Bovie and ligation of perforators with 3-0 vicryl. The rectus muscles were plicated in the midline with figure-of-8 and running O-polydioxanone sutures, without strangulation of the umbilical stalk. Between 2 and 4 #10 Jackson–Pratt drains were placed. The lateral 2 were directed posterior laterally, traversing the superpubic region. The 2 central drains were placed directly over the mid rectus, extending to the undermined area extent of the abdomen. The drains were placed on closed suction. The patient was then elevated to 60° and excess skin was resected. The skin flap was temporarily adjusted and stapled to eliminate dog ears (staples were later replaced with Scarpa's fascia closure using figure-of-8 O-vicryl sutures). The dermis was closed with 3-0 monocryl and running subcuticular 3-0 monocryl sutures. Prineo tapes were used as dressing. The patients were monitored overnight as part of the hospital 23 h stay paradigm, and the Foley catheter was removed on the morning of the first postoperative day. The patients were discharged using same-day surgery criteria. If the treated body surface area exceeded 51% of the body surface area, the overnight stay was strongly recommended by the surgeon for patient convenience; otherwise, the patients returned home.

Standardized postoperative instructions per office protocol were given. Fourteen days of low-dose subcutaneous enoxaparin sodium injection (Lovenox [Winthrop US, A Sanofi company, Bridgewater, NJ]). Abdominal binders were uniformly applied, with initiation at 2 to 7 days postoperatively and continuing for 6 to 8 weeks. Lymphatic massage was utilized throughout this perioperative period. Clinical massages for lymphatic drainage, patient comfort, and soft tissue equilibrium were initiated as soon as 2 days’ postprocedure. Depending on patient preference, these were continued for several weeks perioperatively as a med spa function, and extended massage is not considered integral to outcome. Clinical removal criterion for drains was <30 cc per day for 1 to 2 days before removal.

## RESULTS

### Study Demographics

A total of 40 patients (37 female and 3 male patients, mean age 53.2 years [range, 20-79 years]) treated with lipoabdominoplasty and helium plasma RF and 37 patients (35 female and 2 male patients, mean age 51.8 years [range, 28-87 years]) treated with lipoabdominoplasty alone were included in the analysis. Patient demographics were similar across groups (Table 1), and the groups were well balanced in terms of medical history, including tobacco use and history of massive weight loss (Table 2). Data were not complete for all patients in the analysis, and some variables are reported for less than the total treated population.

**Table 1.** Patient Demographics

Characteristic	Lipoabdominoplasty + helium plasma RF (*n* = 40)	Lipoabdominoplasty (*n* = 37)	*P*-value^a^
Sex, *n*			1.00
Male	3	2	
Female	37	35	
Age (years)			.648
*n*	40	36	
Mean ± SD (min, max)	53.2 ± 13.9 (20, 79)	51.8 ± 11.9 (28, 87)	
BMI (kg/m^2^)			.956
*n*	29	27	
Mean ± SD (min, max)	30.3 ± 7.0 (22.3, 47.9)	30.7 ± 5.4 (22.4, 42.9)	
Fitzpatrick skin type			.147
I-II	19	9	
III-IV	17	22	
V-VI	4	4	

RF, radiofrequency; SD, standard deviation. ^a^Fisher’s exact test for categorical data and *t* test for continuous data.

**Table 2.** Patient Medical History

Characteristic	Lipoabdominoplasty + helium plasma RF (*n* = 40)	Lipoabdominoplasty (*n* = 37)	*P*-value^a^
Tobacco use, *n*			.188
None	20	25	
Current user	4	3	
Previous history	15	7	
Patients reporting births, *n* (%)	20 (50)	22 (59)	.494
Mean (min, max)	2.2 (1, 4)	2.6 (1, 6)	
Patients reporting C-sections, *n* (%)	8 (20)	10 (27)	.592
Mean (min, max)	1.5 (1, 3)	1.9 (1, 4)	
History of massive weight loss, *n* (%)	12 (30)	8 (22)	.445

RF, radiofrequency. ^a^Fisher’s exact test

### Procedure Data

All patients received general anesthesia. The lipoabdominoplasty with the helium plasma RF group had on average more concurrent procedures than the group receiving lipoabdominoplasty alone (average of 1.3 vs 0.7 additional procedures, respectively), and thus longer overall procedure times (Table 3). The lipoabdominoplasty with the helium plasma RF group also had slightly more, although not significantly more, abdominal skin resected (2031 g ± 1578.3 vs 1788 g ± 936.5 for lipoabdominoplasty alone).

**Table 3.** Procedure Data

Characteristic	Lipoabdominoplasty + helium plasma RF (*n* = 40)	Lipoabdominoplasty (*n* = 37)	*P*-value^a^
Blood loss (cc)			.9388
Mean ± SD (min, max)	75.6 ± 35.4 (25, 200)	73.4 ± 176.8 (20, 300)	
Procedure duration (min)			.0008
Mean ± SD (min, max)	444.5 ± 188.8 (237, 617)	318.4 ± 115.3 (153, 580)	
Abdominal fat lipoaspiration (mL)			.0092
Mean ± SD (min, max)	438.5 ± 388.9 (50, 1400)	619.5 ± 139.3 (50, 2000)	
No. of concurrent procedures (*n*)			.0492
Mean ± SD (min, max)	1.05 ± 1.18 (0, 4)	0.59 ± 0.76 (0, 3)	
Drain duration (days)			.8394
Mean ± SD (min, max)	15.5 ± 24.7 (6, 51)	16.4 ± 11.1 (0, 60)	

RF, radiofrequency; SD, standard deviation. ^a^Fisher’s exact test for categorical data and *t* test for continuous data.

For the patients who were treated with helium plasma RF, the average settings were 80% power, 2.4 L/min of helium flow, and 6 passes. Importantly, energy activation was stopped at the time the device tip reached the incision site in order to avoid overheating^17^ at this point of convergence.

### Safety Outcomes: Significant Adverse Events

Serious AEs (SAEs) included abdominal wall cellulitis, tissue necrosis, and wound dehiscence. In the liposuction with helium plasma RF group, 7.5% (3/40) of patients experienced significant AEs that required hospitalization or a home health or ED visit, compared with 8.1% (3/37) of patients who were treated with liposuction alone. A total of 22 patients, 27.5% (11/40) in the helium plasma RF group and 29.7% (11/37) in the lipoabdominoplasty-only group, required an in-office procedure as an intervention to address an AE, including seroma, tissue necrosis, scars, wound dehiscence, and fat cysts. Significant AEs are summarized in Table 4. There were no significant differences between groups. In addition to the absence of a significant difference between the number of patients who experienced SAEs, there was no significant difference between groups regarding the total number of SAEs. The incidence of significant AEs (*P* = .628) was 32.5% (13/40) vs 27.0% (10/37) in the lipoabdominoplasty-only group compared with the helium plasma RF group, suggesting that there is no increased risk associated with adding helium plasma RF to a lipoabdominoplasty procedure.

**Table 4.** Significant Adverse Events Requiring Hospitalization, a Home Health or Emergency Department Visit, or an In-office Procedure

Adverse event	Lipoabdominoplasty + helium plasma RF (*n* = 40) *n* (%)	Lipoabdominoplasty (*n* = 37) *n* (%)	*P*-value^a^
AEs requiring hospitalization or a home health or ED visit			
Abdominal wall cellulitis	1 (2.5)	3 (8.1)	.346
Tissue necrosis	1 (2.5)	—	1.00
Wound dehiscence	1 (2.5)	—	1.00
Total number of patients with 1 or more AE	3 (7.5)	3 (8.1)	1.00
AEs requiring an in-office procedure			
Fat cyst	—	1 (2.7)	1.00
Scar requiring procedure	—	2 (5.4)	1.00
Seroma	6 (15)	5 (13.5)	1.00
Tissue necrosis	1 (2.5)	2 (5.4)	.605
Wound dehiscence	4 (10)	1 (2.7)	.360
Total number of patients with 1 or more SAE	11 (27.5)	11 (29.7)	1.00
Total SAE	13 (32.5)	10 (27.0)	.628

AE, adverse event; ED, emergency department; RF, radiofrequency; SAE, serious adverse event. ^a^Fisher’s exact test.

A univariate model to predict the probability of a significant AE based on only 1 predictor across groups found that for each additional 1000 g of skin excised, the probability of experiencing a significant AE increased on average by 1.3% (95% CI, −6.8%, 9.4%; *P* = .751) and was not a statistically significant predictor. The first multivariate logistic regression model run to predict the probability of a significant AE occurring based on treatment group, age, BMI, history of major weight loss, smoking status, total amount of skin excised, and number of concomitant procedures performed, found no statistically significant predictors of having a significant AE, including treatment type; however, missing data excluded 30 patients from the model. A second multivariate logistic regression model was run excluding BMI and history of major weight loss, and results with only 9 patients excluded also did not identify any strong predictors for patients likely to experience a significant AE, but a univariate model was explored for the number of concomitant procedures, which may be a weak predictor. The relationship between the number of concomitant procedures was not linear with the highest incidence of significant AEs occurring with either 4 or no concomitant procedures. Thus, a third multivariate logistic regression model excluded this confounding predictor and confirmed that there were no significant predictors for experiencing a significant AE. Odds ratios from these 3 models are provided in Table 5.

**Table 5.** Odds Ratios From Multivariate Logistic Regression Models

	Model 1 (*n*^a^ = 47)			Model 2 (*n* = 68)			Model 3 (*n* = 68)		
	Odds ratio	(95% CI)	*P*-value	Odds ratio	(95% CI)	*P*-value	Odds ratio	(95% CI)	*P*-value
Treatment group	2.54	(0.52, 12.48)	.252	1.20	(0.39, 3.76)	.751	0.93	(0.31, 2.79)	.9
Age (years)	1.01	(0.96, 1.07)	.608	1.03	(0.99, 1.07)	.176	1.03	(0.98, 1.07)	.223
BMI	0.98	(0.82, 1.18)	.864						
Significant weight loss	0.28	(0.04, 2.06)	.211						
History of smoking	1.48	(0.33, 6.60)	.604	1.33	(0.43, 4.10)	.614	1.39	(0.47, 4.17)	.553
Amount of skin removed (1000 mL)	1.43	(0.62, 3.32)	.4	1.03	(0.67, 1.58)	.902	1.00	(0.65, 1.54)	.997
No. of procedures	0.65	(0.28, 1.50)	.307	.55	(0.28, 1.10)	.092			

^a^*n* reflects the number of patients included in the model because patients missing any piece of data were excluded.

### Safety Outcomes: Nonsignificant Adverse Events

Outcomes were similar for nonsignificant AEs, occurring in 18% (7/40) of patients who received both liposuction and treatment with helium plasma RF and 8% (3/37) of patients who received liposuction alone. The frequency of individual nonsignificant events for each group is presented in Table 6. Most nonsignificant AEs were experienced by a single patient, with the exception of delayed healing, which was reported in 3 (7.5%) of helium plasma RF patients. Across groups, all significant and nonsignificant events resolved.

**Table 6.** Nonsignificant Adverse Events

Adverse event	Lipoabdominoplasty + helium plasma RF (*n* = 40) *n* (%)	Lipoabdominoplasty (*n* = 37) *n* (%)	*P*-value^a^
Delayed healing	3 (7.5)	1 (2.7)	.616
Fibrosis	1 (2.5)	—	1.00
Granulation of tissue	1 (2.5)	—	1.00
Hematoma	—	1 (2.7)	1.00
Subcutaneous hemorrhage	—	1 (2.7)	1.00
Superficial thrombophlebitis	1 (2.5)	—	1.00
Wound dehiscence	1 (2.5)	—	1.00
Total	7 (17.5)	3 (8.1)	.317

RF, radiofrequency. ^a^Fisher’s exact test.

## DISCUSSION

Overall, this study found that the addition of helium plasma RF to UAL lipoabdominoplasty procedures had no significant effect on the occurrence of significant AEs (*P* = .628). The frequency of significant AEs (requiring hospitalization or an ED or home health visit) was similar: 7.5% (3/40) in the liposuction with helium plasma RF group compared with 8.1% (3/37) for patients who were treated with UAL alone. Similarly, the rate of significant AEs requiring a medical procedure for the helium plasma RF group was 27.5% (11/40) compared with 29.7% (11/37) for patients who were treated with UAL alone. Importantly, the rate of AEs observed here is consistent with those reported in the literature and also occurred primarily in patients with established risk factors.^5,7,22,23^ Although overall complication rates in some UAL studies are as low as 4.6%,^7^ others report that as many as 42% of patients treated with UAL and abdominoplasty developed seromas.^22^ In a study of over 278 patients, 5% had seroma and 2% had delayed healing; however, 24% underwent revisional surgery.^23^ In this same cohort, the seemingly high complication rate was also accompanied by a high level of satisfaction. Because of the differences in how AEs and SAEs are collected across studies, it is difficult to make a comparison, particularly because some rates seem to be higher than what is observed in clinical practice. In this study, there were no revisions; however, 11 of 40 patients (27.5%) in the helium plasma RF group had an AE requiring medical attention (compared with 29.7% [11/37] for lipoabdominoplasty alone). The rate of seroma is within the reported range, with 6/40 (15%) and 5/37 (13.5%) in the helium plasma RF and lipoabdominoplasty-alone groups, respectively.

The safe addition of liposuction to abdominoplasty has been demonstrated by several retrospective studies. In one retrospective study of 300 cases, there was no increase in complication rates observed for cases in which a perforator vessel was spared,^24^ confirming earlier studies in which no difference was shown in safety outcomes for abdominoplasty with liposuction compared with liposuction alone.^25^ The lipoabdominoplasty technique described here has been characterized by Matarasso, who based his vascular analysis of work by Huger.^25,26^ The Type IV abdominoplasty surgical elevation performed in these patients adheres to the Lockwood recommendation against aggressive liposuction, because this technique does not employ central high-abdomen barotrauma from nonselective suction cannulae.^27^ Importantly, UAL lipoabdominoplasty has been shown in larger retrospective studies of >600 patients to be a safe adjunct to abdominoplasty, and the application of UAL in this setting is associated with significantly less blood loss in addition to improved skin contraction.^5,28^ Further, the addition of helium plasma RF to liposuction has been shown to be safe.^29^ Of note, in 1 retrospective study, seroma was observed in patients who received both liposuction and helium plasma RF (with 6.8% of patients reporting 17 events) but not in patients who received helium plasma RF alone. In this study, 15% of patients experienced seroma in the lipoabdominoplasty with helium plasma RF group compared with 13.5% of patients in the lipoabdominoplasty-alone group. Taken together, in this study, safety equivalence in using high energy and the duration of ultrasound energy without suction when directed to the high Huger zones are suggested. The use of helium plasma RF energy in the same higher Huger zones also appears to be safe.

Together, the authors of these studies, along with the one described here, support the application of UAL for lipoabdominoplasty, but also support the hypothesis that the addition of thermal energy and tissue coagulation with helium plasma RF following liposuction does not compromise the skin flap or carry additional risk.^29^ In the present study, helium plasma RF was shown to not be associated with additional AEs, supporting its use in the setting of lipoabdominoplasty.

Multivariate analysis did not identify any other risk factors that would make a patient more prone to AEs with or without the addition of helium plasma RF when applied to the high Huger Zones I and II. Univariate modeling did suggest that the volume of skin excised could impact AE risk; however, this finding was not particularly compelling because of patient variability in skin volume excised. Of note, smoking was not identified as a risk factor, but this was likely because of the relatively small number of patients in the study who were active smokers. Critically, there was no association identified between surgical time and AEs.

Although the study described here includes a small number of patients, clinically these results indicate that helium plasma RF may be a safe adjunct for lipoabdominoplasty as performed by the authors, because there do not appear to be additional complications associated with treating Huger zones with both UAL and helium plasma RF with lipoabdominoplasty.^25,26^ Importantly, the safety of helium plasma RF in this setting also lends support for use in other areas in which there is concern for the surgical flap, because in the findings here, no difference is shown in safety signals between the treatment groups. Importantly, because the rate of AEs was low overall, a prohibitively large study would be needed to detect statistically significant differences between groups, highlighting the low rate of AEs for both groups. In the findings, the additional heat introduced by helium plasma RF does not negatively affect the abdominal flap and does not affect important outcomes such as the amount of time a drain is needed. Of note, in spite of the fact that the helium plasma RF group had more concomitant procedures and longer surgical times, there was no increase in significant or nonsignificant AEs. Taken together this study’s findings support the safe incorporation of helium-based plasma RF in this area. This retrospective, single-surgeon study is the first of its kind in which UAL lipoabdominoplasty is compared with UAL lipoabdominoplasty with helium plasma RF. In the author's clinical experience, in addition to tissue contraction, helium plasma RF in the abdomen results in the same improvement in fine wrinkling, evenness in skin tone, and harmony in contour observed with treatment in the submentum and observed by the author in clinical practice for the face.^29^ It will be important to fully and carefully characterize these types of changes not only in the face and abdomen, but also in other body areas treated with UAL, such as the arms, thighs, and buttocks, among others.

The limitations of the current study include the retrospective design, single-surgeon design, and the small patient number. For example, smoking status, an established risk factor, was not identified as such, likely because of the small subset of patients who were active smokers. Even so, this study does include real-world data from 77 patients and suggests that the addition of helium plasma RF to UAL abdominoplasty may not pose an increased safety risk. A larger, registry-style study could be done by researchers to further assess safety in a larger group of patients. Further, in this study, efficacy was not assessed, and additional studies could be designed by researchers to better define aesthetic benefit, which would be an important complement to these safety data. In addition, the patients were treated over the course of 5.5 years, wherein the single surgeon improved his technique and efficiency of implementation.

## CONCLUSIONS

In this study, it is indicated that the helium plasma RF device may be safe as an adjunct for UAL lipoabdominoplasty. When used for subdermal coagulation in the abdominal area, the device does not appear to increase the incidence of SAEs or introduce risk to the flap.

## References

[1] The Aesthetic Society. Aesthetic Plastic Surgery National Databank: 2022. Accessed March 8, 2024. https://cdn.theaestheticsociety.org/media/statistics/2022-TheAestheticSocietyStatistics.pdf

[2] Shauly O, Goel P, Gould DJ. Painless, drainless lipoabdominoplasty: a retrospective study of pain following lipoabdominoplasty utilizing liposomal bupivacaine and a modified enhanced recovery after surgery protocol. Aesthet Surg J Open Forum. 2022;4:ojac049. doi: 10.1093/asjof/ojac049 35854876 PMC9280521

[3] Hurwitz D, Smith D. Treatment of overweight patients by radiofrequency-assisted liposuction (RFAL) for aesthetic reshaping and skin tightening. Aesthet Plast Surg. 2012;36:62–71. doi: 10.1007/s00266-011-9783-z 21751063

[4] Chia CT, Theodorou SJ. 1,000 consecutive cases of laser-assisted liposuction and suction-assisted lipectomy managed with local anesthesia. Aesthet Plast Surg. 2012;36:795–802. doi: 10.1007/s00266-012-9885-2 22447150

[5] Nagy MW, Vanek PF Jr. A multicenter, prospective, randomized, single-blind, controlled clinical trial comparing VASER-assisted lipoplasty and suction-assisted lipoplasty. Plast Reconstr Surg. 2012;129:681e–689e. doi: 10.1097/PRS.0b013e3182442274 22456382

[6] Troell RJ. Lipoabdominoplasty: comparing ultrasound-assisted and power-assisted techniques. Am J Cosm Surg. 2023;40:279–292. doi: 10.1177/07488068221099153

[7] Tran BNN, Didzbalis CJ, Chen T, Shulzhenko NO, Asaadi M. Safety and efficacy of third-generation ultrasound-assisted liposuction: a series of 261 cases. Aesthet Plast Surg. 2022;46:2310–2318. doi: 10.1007/s00266-022-02992-7 35896731

[8] Wall SH Jr, Claiborne JR. Discussion: a report of 736 high-definition lipoabdominoplasties performed in conjunction with circumferential VASER liposuction. Plast Reconstr Surg. 2018;142:676–678. doi: 10.1097/PRS.0000000000004711 29878994

[9] US Food and Drug Administration. 510(k) summary for InMode RF System. K151793. Accessed October 4, 2022. https://www.accessdata.fda.gov/cdrh_docs/pdf15/K151793.pdf

[10] Key DJ. Integration of thermal imaging with subsurface radiofrequency thermistor heating for the purpose of skin tightening and contour improvement: a retrospective review of clinical efficacy. J Drugs Dermatol. 2014;13:1485–1489. 25607794

[11] Chia CT, Theodorou SJ, Hoyos AE, Pitman GH. Radiofrequency-assisted liposuction compared with aggressive superficial, subdermal liposuction of the arms: a bilateral quantitative comparison. Plast Reconstr Surg Glob Open. 2015;3:e459. doi: 10.1097/GOX.0000000000000429 26301148 PMC4527633

[12] Duncan DI. Improving outcomes in upper arm liposuction: adding radiofrequency-assisted liposuction to induce skin contraction. Aesthet Surg J. 2012;32:84–95. doi: 10.1177/1090820X11429549 22231416

[13] Paul M, Blugerman G, Kreindel M, Mulholland RS. Three-dimensional radiofrequency tissue tightening: a proposed mechanism and applications for body contouring. Aesthet Plast Surg. 2011;35:87–95. doi: 10.1007/s00266-010-9564-0 PMC3036829 20835826

[14] Irvine Duncan D. Nonexcisional tissue tightening: creating skin surface area reduction during abdominal liposuction by adding radiofrequency heating. Aesthet Surg J. 2013;33:1154–1166. doi: 10.1177/1090820X13505862 24335016

[15] DiBernardo BE, Reyes J. Evaluation of skin tightening after laser-assisted liposuction. Aesthet Surg J. 2009;29:400–407. doi: 10.1016/j.asj.2009.08.006 19825469

[16] Han X, Yang M, Yin B, et al The efficacy and safety of subcutaneous radiofrequency after liposuction: a new application for face and neck skin tightening. Aesthet Surg J. 2021;41:NP94–NP100. doi: 10.1093/asj/sjz364 32004377

[17] Irvine Duncan D, Roman S. Helium plasma subdermal tissue contraction method of action. BioMed J Sci Technol Res. 2020;31:24063–24068. doi: 10.26717/BJSTR.2020.31.005075

[18] US Food and Drug Administration. 510(k) summary for Apyx Plasma/RF Handpiece. K191542. Accessed February 1, 2024. https://www.accessdata.fda.gov/cdrh_docs/pdf19/K191542.pdf

[19] US Food and Drug Administration. 510(k) summary for VASERlipo System. K190551. Accessed March 8, 2024. https://www.accessdata.fda.gov/cdrh_docs/pdf19/K190551.pdf

[20] Wright NT, Humphrey JD. Denaturation of collagen via heating: an irreversible rate process. Annu Rev Biomed Eng. 2002;4:109–128. doi: 10.1146/annurev.bioeng.4.101001.131546 12117753

[21] Chen SS, Humphrey JD. Heat-induced changes in the mechanics of a collagenous tissue: pseudoelastic behavior at 37 degrees C. J Biomech. 1997;31:211–216. doi: 10.1016/s0021-9290(97)00121-8 9645535

[22] Kim J, Stevenson TR. Abdominoplasty, liposuction of the flanks, and obesity: analyzing risk factors for seroma formation. Plast Reconstr Surg. 2006;117:773–779; discussion 780-1. doi: 10.1097/01.prs.0000200056.57357.3f 16525264

[23] Stewart KJ, Stewart DA, Coghlan B, Harrison DH, Jones BM, Waterhouse N. Complications of 278 consecutive abdominoplasties. J Plast Reconstr Aesthet Surg. 2006;59:1152–1155. doi: 10.1016/j.bjps.2005.12.060 17046623

[24] Smith LF Jr, Smith LF. Safely combining abdominoplasty with aggressive abdominal liposuction based on perforator vessels: technique and a review of 300 consecutive cases. Plast Reconstr Surg. 2015;135:1357–1366. doi: 10.1097/PRS.0000000000001200 25919250 PMC4410962

[25] Matarasso A. Liposuction as an adjunct to a full abdominoplasty. Plast Reconstr Surg. 1995;95:829–836. doi: 10.1097/00006534-199504001-00010 7708866

[26] Huger WE Jr. The anatomic rationale for abdominal lipectomy. Am Surg. 1979;45:612–617. 159651

[27] Lockwood T. High-lateral-tension abdominoplasty with superficial fascial system suspension. Plast Reconstr Surg. 1995;96:603–615. doi: 10.1097/00006534-199509000-00012 7638284

[28] Hoyos A, Perez ME, Guarin DE, Montenegro A. A report of 736 high-definition lipoabdominoplasties performed in conjunction with circumferential VASER liposuction. Plast Reconstr Surg. 2018;142:662–675. doi: 10.1097/PRS.0000000000004705 29878992

[29] Ruff PG, Vanek P, Nykiel M. Adverse events of soft tissue coagulation using a helium-based plasma technology alone and in combination with ultrasound-assisted liposuction. Aesthet Surg J Open Forum. 2022;4:ojac064. doi: 10.1093/asjof/ojac064 36211477 PMC9536283

